# HIV-1 Gag Polyprotein Affinity to the Lipid Membrane Is Independent of Its Surface Charge

**DOI:** 10.3390/biom14091086

**Published:** 2024-08-29

**Authors:** Zaret G. Denieva, Valerij S. Sokolov, Oleg V. Batishchev

**Affiliations:** Laboratory of Bioelectrochemistry, Frumkin Institute of Physical Chemistry and Electrochemistry, Russian Academy of Sciences, 119071 Moscow, Russia; zaret03@mail.ru (Z.G.D.); sokolov.valerij@gmail.com (V.S.S.)

**Keywords:** human immunodeficiency virus (HIV), Gag polyprotein, bilayer lipid membrane, cholesterol, membrane charge, inner field compensation method, boundary potential, ζ-potential, surface potential, binding constant

## Abstract

The binding of the HIV-1 Gag polyprotein to the plasma membrane is a critical step in viral replication. The association with membranes depends on the lipid composition, but its mechanisms remain unclear. Here, we report the binding of non-myristoylated Gag to lipid membranes of different lipid compositions to dissect the influence of each component. We tested the contribution of phosphatidylserine, PI(4,5)P2, and cholesterol to membrane charge density and Gag affinity to membranes. Taking into account the influence of the membrane surface potential, we quantitatively characterized the adsorption of the protein onto model lipid membranes. The obtained Gag binding constants appeared to be the same regardless of the membrane charge. Furthermore, Gag adsorbed on uncharged membranes, suggesting a contribution of hydrophobic forces to the protein–lipid interaction. Charge–charge interactions resulted in an increase in protein concentration near the membrane surface. Lipid-specific interactions were observed in the presence of cholesterol, resulting in a two-fold increase in binding constants. The combination of cholesterol with PI(4,5)P2 showed cooperative effects on protein adsorption. Thus, we suggest that the affinity of Gag to lipid membranes results from a combination of electrostatic attraction to acidic lipids, providing different protein concentrations near the membrane surface, and specific hydrophobic interactions.

## 1. Introduction

The Gag polyprotein is the major component of the human immunodeficiency virus (HIV-1). It consists of several domains, including the N-terminally myristoylated matrix (MA), capsid (CA), nucleocapsid (NC), and p6 domains, and two linker peptides, SP1 and SP2 [1]. Gag is synthesized in the cytoplasm, translocates to the cell periphery, and ultimately targets the surface of the plasma membrane (PM), where immature virus assembly occurs [2]. Studies of the truncated Gag-Δp6 protein (lacking myristoyl and p6 domain) show its ability to form virus-like particles even in the cytoplasm [3]. On the other hand, these particles are often defective in the absence of additional factors like the presence of inositol phosphates IP5, IP6, or RNA [4,5,6]. Solution studies suggest that Gag forms only low-order oligomers like dimers or trimers [7,8]. Simultaneously, fluorescence resonance energy transfer experiments suggest that Gag multimerization occurs only at the plasma membrane [9,10,11].

The MA domain of Gag is thought to be the structural motif that mediates targeting to the PM [12]. Interaction with the PM is mediated by electrostatic attraction between the highly basic region (HBR) of the MA domain and anionic membrane lipids, as well as by specific binding of the protein to phosphatidylinositol 4,5-bisphosphate (PI(4,5)P2) [13]. This lipid is a low abundant (1–2 mol%) phospholipid found in the cytoplasmic leaflet of the PM [14]. The specificity of the interaction of Gag with PI(4,5)P2 is explained by two mechanisms: (i) It acts as an “anchor”, fixing the protein in the lipid bilayer by electrostatic interactions, and (ii) it triggers the release of myristoyl from its sequestered state within the protein, which holds Gag in the membrane by hydrophobic forces [15]. However, MA without the myristoyl moiety could still bind to membranes [16]. Mutations in the HBR of the MA domain suppress Gag–membrane binding in cells in the absence of PI(4,5)P2 [17]. Thus, the interactions between Gag and PM are thought to be mainly electrostatic.

Studies of Gag–membrane interactions often point to the specific role of PI(4,5)P2. However, the major anionic lipid in the cell plasma membrane is phosphatidylserine (PS). Since PI(4,5)P2 and PS are both negatively charged, it remains unclear whether the surface charge of phosphatidylserine might be sufficient for Gag–membrane binding [18]. However, there are few data on the adsorption of HIV-1 Gag protein onto lipid membranes with phosphatidylserine without PI(4,5)P2. Some work has been done only on single MA domains [16,19] or on Gag proteins of other retroviruses [20,21,22].

In addition to the MA domain, the NC domain of HIV-1 Gag plays an important role in membrane interactions, as Gag containing this domain has been found to be more prominently distributed in the PM [23]. Although the primary role of NC is to bind to RNA, resulting in Gag oligomerization, a non-exclusive hypothesis is that NC may be involved in the binding of Gag to the PM. Deletion of NC or replacement of all its basic residues with neutral ones has been reported to partially affect the anchoring and multimerization of Gag at the PM [10,24]. A strong interaction of NC with negatively charged lipid membranes, both in its free form and bound to nucleic acids, is demonstrated by a combination of fluorescence spectroscopy techniques [25]. It is found that the number of NC binding sites, but not the binding constant, decreases with the percentage of negatively charged lipids in the membrane, suggesting that NC and its complex with nucleic acid are able to recruit negatively charged lipids to ensure optimal binding. However, unlike MA, NC does not show a preference for PI(4,5)P2.

Lipidomic studies have shown that raft-associated lipids, such as cholesterol, are enriched in the envelope of HIV-1 [26,27]. It has been shown to strongly influence Gag–membrane binding [28]. Cholesterol leads to a tighter packing of phospholipids, and a combination of PI(4,5)P2 with cholesterol enhances Gag binding in an additive manner [28]. Gag has been reported to limit the mobility of PI(4,5)P2 and cholesterol in the cell plasma membrane [29]. This could be explained by the Gag-induced stabilization of domains formed by phosphoinositides due to the intermolecular hydrogen bond network formed between the lipid headgroups [30]. The intra- and intermolecular hydrogen bond network between the phosphoinositide lipids leads to a reduction in charge density in the phosphoinositide phosphomonoester groups [31]. Therefore, cholesterol acts as a spacer between the phosphoinositide lipids, thereby reducing electrostatic repulsion, as well as participating in the hydrogen bond network, leading to its further stabilization. In contrast, molecular dynamics simulations show a large cholesterol-driven increase in membrane surface charge density as a result of lipid packing [32]. This makes the electrostatic potential at the membrane surface more negative, which affects protein association with the membrane.

The electric field generated by acidic lipids at the membrane/water interface generally consists of two parts [33]. One is a surface potential, which is the potential drop in the diffuse part of the electrical double layer (EDL) between the membrane surface and the bulk electrolyte. The other part is the dipole potential associated with the dipoles of lipid head groups and bound water molecules. The sum of surface and dipole potentials gives the total jump in electrical potential, the boundary potential, which changes during protein adsorption onto the membrane. Thus, changes in these potentials should reflect Gag–membrane interactions. Recently, using the inner field compensation (IFC) method [34], we have shown that the binding of non-myristoylated HIV-1 Gag-Δp6 (hereafter referred to as Gag) to PS-containing and uncharged membranes occurs with the same intrinsic binding constants [35].

Here we studied Gag–membrane interactions by systematically varying the lipid composition of the membranes. We determined the contributions of acidic lipids (PS and PI(4,5)P2) and cholesterol to both membrane surface potential and Gag affinity. We clearly showed that the apparent differences in Gag adsorption onto membranes of different charges were determined by the difference in protein surface concentration near the lipid membrane. Only the presence of PI(4,5)P2 in combination with cholesterol led to specific protein–lipid interactions, changing the adsorption mode and showing cooperative effects.

## 2. Materials and Methods

### 2.1. Materials

Buffer solutions were prepared using KCl (Sigma-Aldrich, Saint-Louis, MO, USA), HEPES (Helicon, Moscow, Russia), EDTA (Life Technologies, Carlsbad, CA, USA), KOH (Reachem, Moscow, Russia), HCl (Reachem, Moscow, Russia), and agar (Helicon, Moscow, Russia). Membranes were formed in n-decane (Acros Organics, Geel, Belgium) using the following stock solutions of lipids: 1,2-diphytanoyl-sn-glycero-3-phosphocholine (DPhPC), 1,2-diphytanoyl-sn-glycero-3-phosphoserine (DPhPS), phosphatidylinositol-4,5-bisphosphate (PI(4,5)P2), and cholesterol (Chol) (all from Avanti Polar Lipids, Alabaster, AL, USA) dissolved in chloroform (99%, Merck, Darmstadt, Germany) at the concentration of 10 mg/mL.

### 2.2. Protein Purification

Recombinant HIV-1 Gag was purified after expression in *E. coli* BL21 (DE3) pLysS, essentially as described in detail previously [3]. Briefly, the protein was overexpressed at 37 °C for 4 h with 0.4 mM IPTG. The cell lysate was treated with 0.1% (*w/v*) polyethylene imine, to eliminate nucleic acids, and was subsequently subjected to ammonium sulfate fractionation (30% saturation). The pellet enriched for Gag was affinity-purified using phosphocellulose resin, which binds the NC domain of Gag with high affinity. The protein at this stage has ~85% purity. The protein was further subjected to size exclusion chromatography, on a Superose-12 HPLC column equilibrated with 0.5 M NaCl, 20 mM HEPES, 2 mM DTT, 10% *w/v* glycerol, and pH 7.4. Peak fractions containing protein were then concentrated to ~5 mg/mL, aliquoted, and stored at −80 °C. At this stage, the purity of the protein (>94% pure) was accessed by SDS PAGE and highly sensitive “Blue Silver” staining [36]. The protein lacks the myristoyl group and p6 domain.

### 2.3. Model Lipid Membranes

Flat bilayer lipid membranes (BLMs) were formed by the Müller–Rudin technique at the 1 mm diameter circular aperture in a partition separating two compartments of a Teflon chamber [37]. Each compartment contained the working buffer solution of 10 mM KCl, 5 mM HEPES, 0.1 mM EDTA, and pH 7.2 in Milli-Q water. Electrical measurements were made using Ag/AgCl electrodes placed in each compartment in contact with the working buffer via salt bridges (2% agar in 100 mM KCl solution). Salt bridges were used to attain the resistance of the electrodes < 50 Ohms. Membranes were formed from a lipid solution with a concentration of 15 mg/mL in n-decane. For electrical measurements, an AC voltage generator (output of the L780 DAC board, Lcard, Moscow, Russia) was connected to the electrode at one side of the BLM, and a current amplifier “Keithley-427” (Keithley, Solon, OH, USA) was connected to the electrode at the other side. The formation of a BLM was registered by the appearance of the capacitance of 1–3 nF, assuming that the membrane occupied the entire area of the aperture. A magnetic stirrer was used to mix the buffer solution in the chamber during the experiment in order to maintain the same kinetics of adsorption.

Liposomes were prepared by the lipid film hydration method [38]. Briefly, a thin lipid film was obtained at the bottom of a round-bottomed glass flask by evaporation of a lipid solution in chloroform (at a concentration of 10 mg/mL) on a rotary evaporator (40 min at 40 mbar pressure). The film was then hydrated with a working buffer and vortexed on a BioVortex (Bio-San, Riga, Latvia) to obtain the liposomes with a final concentration of 1 mg/mL.

### 2.4. Inner Field Compensation Method

The boundary potential difference (Δφ_b_) of the BLM was measured by the IFC method, which uses the second harmonic of the capacitance current to measure the membrane boundary potential difference [34]. The method exploits the ability of the membrane to reduce its thickness when an electric field is applied, thereby increasing its electrical capacitance. The capacitance reaches a minimum when the intramembrane field is zero and the corresponding voltage between water solutions is equal to the difference of the boundary potentials across the membrane. This voltage can be found by measuring the second harmonic amplitude of the capacitance current with the phase-sensitive amplifier (Stanford DSP lock-in amplifier, model SR830, Sunnyvale, CA, USA) while applying a sinusoidal voltage with a DC bias to the membrane. The DC voltage corresponding to the minimum membrane capacitance is determined as the DC bias at which the second harmonic of the capacitance current becomes zero. The setup continuously maintains the second harmonic at zero by negative feedback and records the difference in membrane boundary potentials as a function of time. The method allows measuring the change in the boundary potential due to the asymmetric (one-sided) adsorption of charged molecules on the membrane. The difference in the boundary potentials was measured for different bulk concentrations of Gag added to one of the two compartments of the experimental chamber. In the experiments, the bulk electrolyte with low ionic strength (10 mM KCl, 5 mM HEPES, 0.1 mM EDTA) was used to reduce the screening effects of the electrolyte ions and thus increase the resolution of the IFC method [39].

### 2.5. ζ-Potential

Electrokinetic measurements of the electric potential in the hydrodynamic slipping plane (ζ-potential) in the suspension of liposomes were performed to obtain the surface potential of the membranes [40]. The electrophoretic mobility of liposomes was determined by the dynamic light scattering method using a Zetasizer II device (Malvern Instruments, Malvern, Worcestershire, UK) with the PhotoCor SP correlator (PhotoCor, Moscow, Russia). A voltage of 100–120 V was applied to create the electric field between two platinum electrodes located 5 cm apart in the electrophoretic cell. The electrodes were separated from the sample by a membrane impermeable to colloidal particles. To avoid polarization of the electrodes, the polarity of the potential applied to the electrodes was changed at a frequency of 2 Hz. The ζ-potential can be obtained using the Smoluchowski equation [40]. All measurements were performed under low ionic strength conditions of the bulk electrolyte (10 mM KCl, 5 mM HEPES, 0.1 mM EDTA, pH 7.2) in Milli-Q water.

## 3. Results

### 3.1. Charge Density on Lipid Membranes

It is generally accepted that the polyprotein Gag binds to the cell plasma membrane for the assembly of immature viruses. The inner leaflet of the plasma membrane contains various acidic phospholipids; thus, electrical double layers are formed near the surface of the membrane. The Gag polyprotein is thought to interact with membrane regions enriched in negatively charged lipids, such as phosphatidylserine (PS) and phosphatidylinositol 4,5-bisphosphate (PI(4,5)P2) [41,42,43]. In addition to acid lipids, cholesterol plays an important role in protein adsorption [28,30]. Here, we used different lipid mixtures to form membranes and determined the contribution of each lipid to the surface potential at the membrane/water interface. The lipid compositions were chosen to replicate the major lipid components of the site where viral assembly is initiated, i.e., the inner leaflet of the plasma membrane. However, because phosphatidylethanolamine, present at high concentrations in the inner leaflet of the PM, did not form stable flat BLMs, we used phosphatidylcholine (PC) as the major component of the lipid bilayers. We also investigated the role of phosphatidylserine (DPhPS), PI(4,5)P2, and cholesterol (Chol). BLMs were prepared from the following lipid mixtures:

DPhPC = 100 mol% (Mixture 1)

DPhPC:DPhPS = 80:20 mol% (Mixture 2)

DPhPC:PI(4,5)P2 = 98:2 mol% (Mixture 3)

DPhPC:DPhPS:PI(4,5)P2 = 78:20:2 mol% (Mixture 4)

DPhPC:DPhPS:Chol = 50:20:30 mol% (Mixture 5)

DPhPC:DPhPS:PI(4,5)P2:Chol = 48:20:2:30 mol% (Mixture 6)

Electrochemical methods allow the measurement of average electrical potentials in planes, close to the surface of the lipid membrane. The classical Gouy–Chapman–Stern (GCS) model describes well the experimental data for several lipid models of biological membranes [44]. The relationship between the surface potential, φ(0), and the surface charge density, $\sigma_{0}$, is given by the Equation (1). The potential distribution, φ(x), near the charged surface is described by the Equation (2). The ion concentration near the membrane surface is described by the Boltzmann distribution (3). The adsorption of cations onto negatively charged phospholipids is described by the simple Langmuir isotherm (4).

(1) $$\sigma_{0}=\sqrt{8RT\varepsilon \varepsilon_{0}C_{bulk}}sinh\left(\frac{zF\varphi \left(0\right)}{2RT}\right),$$

(2) $$tanh\left(\frac{z_{i}F\varphi \left(x\right)}{4RT}\right)=\exp\left(-\kappa x\right)tanh\left(\frac{z_{i}F\varphi \left(0\right)}{4RT}\right),$$

(3) $$C\left(0\right)=C_{bulk}\exp\left(-\frac{z_{i}F\varphi \left(0\right)}{RT}\right),$$

(4) $$\frac{\sigma_{0}}{\sigma_{max}}=\frac{1}{1+K_{El}C(0)},$$

where $\sigma_{0}$ is the surface charge density, $\sigma_{max}$ is the maximal surface charge density of anionic phospholipids, φ(*x*) is the electric potential at the distance x from the membrane surface, φ(0) is the membrane surface potential, $\kappa =\sqrt{\frac{2z^{2}F^{2}C_{bulk}}{\varepsilon \varepsilon_{0}RT}}$ is the reverse Debye screening length, C_bulk_ is the concentration of bulk electrolyte ions, C(0) is the concentration of electrolyte ions at the membrane surface, K_El_ is the equilibrium constant of the cation adsorption, z_i_ is the charge number of electrolyte ions, ε and ε_0_ are dielectric constants in the solution and in vacuum, respectively, T is the absolute temperature, R is the gas constant, and F is the Faraday constant.

**Figure 1:**
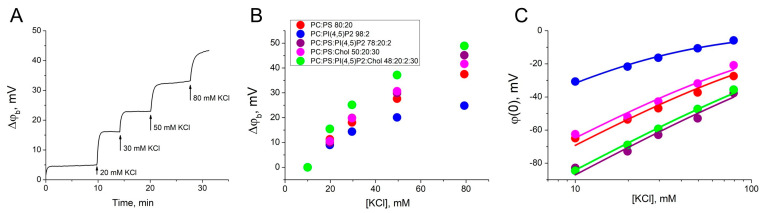
Determination of the charge density on the surface of the BLMs. (**A**) Example of changes of the Δφ_b_ at the BLM of mixture 2 as a result of the increase of electrolyte ionic strength by 1 M KCl addition to the one side of the membrane. (**B**) The growth of the Δφ_b_ at the BLM as a function of the KCl concentration. In the legend, ratios are given in mol%. (**C**) The surface potential at the BLM as a function of the KCl concentration. The approximation curves were plotted using Equations (1)–(4). Each point in the plots was obtained by averaging the results of 3–5 independent experiments. Error bars in the plots represent the standard deviation. If the error bars are smaller than the size of the circle in the plot, the error bars are not shown.

First, we obtained a dependence of the difference in boundary potentials (Δφ_b_) on the ionic strength of the bulk electrolyte to measure the surface charge density and the binding constant of bulk potassium cations. The membrane of each charged lipid mixture (2–6) was formed in a 10 mM KCl, and the ionic strength of the bulk electrolyte on the one side of the BLM was sequentially increased the by the addition of the 1 M KCl. During this procedure, the Δφ_b_ was recorded using the IFC method (Figure 1A). The value of the difference in boundary potentials for each concentration of KCl is the sum of the effects of all previous additions (Figure 1B). Zero corresponded to the surface potential at 10 mM KCl obtained from electrokinetic measurements of ζ-potential in the hydrodynamic slipping plane (Table 1). The distance x from the membrane surface to the slipping plane, determined as 0.2 nm for lipid membranes, is commonly used for the analysis of experimental data [45,46]. This parameter allows good agreement between the electrokinetic data and data obtained on planar BLMs [33]. Using this, ζ-potential values were recalculated into the surface potentials at the BLM, φ(0) (Figure 1C). The data obtained were approximated by Equations (1)–(4) to define the surface charge density at the membrane (Table 1). For lipid mixtures 2–6 the potassium ion binding constant (K_El_) was almost the same and the highest value was determined to be 1.0 ± 0.1 M^−1^, which is in agreement with the value obtained from the ζ-potential measurements [45,47]. This value is quite small and can be neglected when calculating the constants for the Gag adsorption. Potassium ions do not bind to uncharged membranes of mixture 1, as has been demonstrated previously [35,45]. Our results are in agreement with those reported in the literature for liposomes containing acidic lipids [44,47,48,49].

### 3.2. HIV-1 Gag Polyprotein Adsorption onto Lipid Membranes

**Figure 2:**
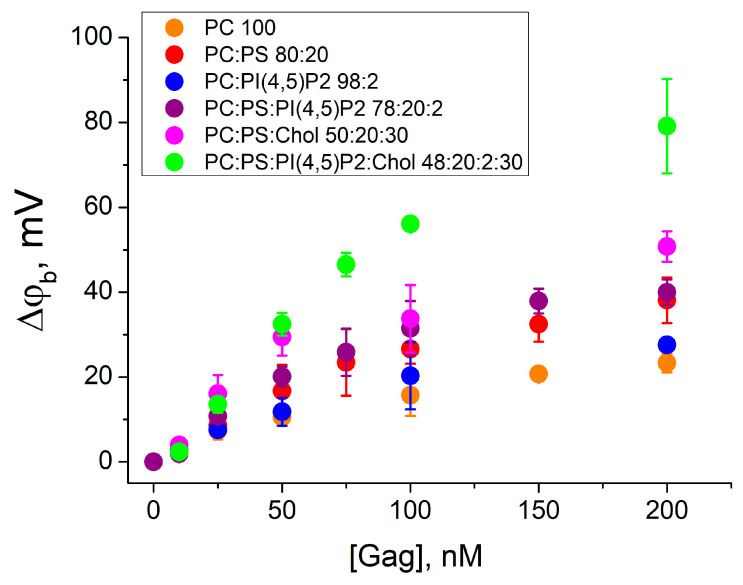
The difference in boundary potentials measured after addition of Gag to the one side of the membrane. BLMs were formed from the mixtures 1–6 (in the legend ratios are given in mol%). Each point in the plots was obtained by averaging the results of 3–5 independent experiments. Error bars in the plots represent the standard deviation. If the error bars are smaller than the size of the circle in the plot, the error bars are not shown.

The kinetics of Gag adsorption onto the BLM was studied by the IFC method [34]. Gag was added asymmetrically (to the one side) to lipid membranes of different compositions (mixtures 1–6), and the difference of boundary potentials across the membrane was recorded as a function of the bulk protein concentration (C_Gag_) (Figure 2). The Δφ_b_ value reached the steady state approximately 15 min after the protein addition. The Gag concentration varied from 10 to 200 nM, which is within the biologically relevant range of protein concentrations in the cytoplasm of cells (up to 500–700 nM [50]). In our previous work, we have shown that Gag at concentrations of approximately 300 nM and higher decreases membrane lateral tension [51]. Therefore, in order to accurately evaluate protein adsorption rather than changes in membrane properties, we used Gag concentrations no higher than 200 nM. In the experiments, the protein was added to reach the final bulk concentration, and the Δφ_b_ values were obtained independently for each Gag concentration. Each point in the plots in Figure 2 was obtained by averaging the results of 3–5 independent experiments. Error bars in the plots represent the standard deviation. If the error bars were smaller than the size of the circle in the plot, the error bars were not shown.

The addition of Gag to the zwitterionic DPhPC membrane (mixture 1) resulted in an increase in the difference of boundary potentials from 3 ± 2 mV to 23 ± 2 mV with increasing bulk Gag concentrations. This behavior indicates the fundamental possibility of the Gag adsorption on an uncharged lipid membrane, as we have previously demonstrated [35]. A positive change in Δφ_b_ corresponds to the presence of positive charges at the membrane/water interface. Thus, in the case of uncharged membranes, Gag presumably orients its basic amino acid residues towards the aqueous phase and binds to the membrane through hydrophobic interactions.

The effect of the negatively charged phosphatidylserine was investigated by the Gag adsorption onto a BLM of the mixture 2. The presence of 20 mol% DPhPS in the membrane enhanced the adsorption of Gag, as indicated by higher positive values of Δφ_b_. The change in the Δφ_b_ was from 3 ± 1 mV to 38 ± 5 mV, which is two times higher compared to the uncharged mixture 1. However, as we have shown in [35], this growth only reflects the increase in the surface concentration of the positively charged Gag near the negatively charged membrane. The contribution of PI(4,5)P2 to the electrostatic attraction and specific protein–lipid interactions was investigated for the BLM of mixture 3 containing 2 mol% of this lipid. The change in the difference of the boundary potential difference was from 3 ± 1 mV to 28 ± 2 mV with increasing Gag concentration, as for mixture 1. However, it can be seen that the difference in surface potential (Table 1) for mixtures 1 and 3 is also about 27 mV. Therefore, the surface concentration of Gag molecules near the membrane was higher for mixture 3 comparing mixture 1. The combination of DPhPS and PI(4,5)P2 in the BLM showed that in this case 2 mol% of PI(4,5)P2 had practically no effect on the interaction of Gag with the membrane. Adsorption of Gag onto the BLM of mixture 4 resulted in an increase in the measured difference of the boundary potentials from 1.9 ± 0.3 mV to 40 ± 3 mV with increasing protein concentration, which is the same as for mixture 2 without PI(4,5)P2. These results are consistent with previous data obtained for the MA domain alone and for N-terminal peptides [19,52,53,54,55,56].

The presence of 30 mol% cholesterol in lipid membranes resulted in higher values of Δφ_b_ during Gag adsorption. The addition of Gag in concentrations from 25 nM to 200 nM to the membrane of the mixture 5 resulted in the difference of boundary potentials from 16 ± 4 mV to 51 ± 4 mV. This is about 30% higher than in mixture 2. A greater increase in the difference of the difference of boundary potentials was observed upon adsorption of Gag onto membranes containing Chol and both acidic lipids (mixture 6). The amplitude of the Δφ_b_ values changed from 2 ± 1 mV to 79 ± 11 mV with an increase in Gag concentration from 10 nM to 200 nM. This is two-fold higher than the values measured for the membrane without cholesterol (mixture 4). These results confirm that Gag can sense cholesterol, as reported previously [28].

**Figure 3:**
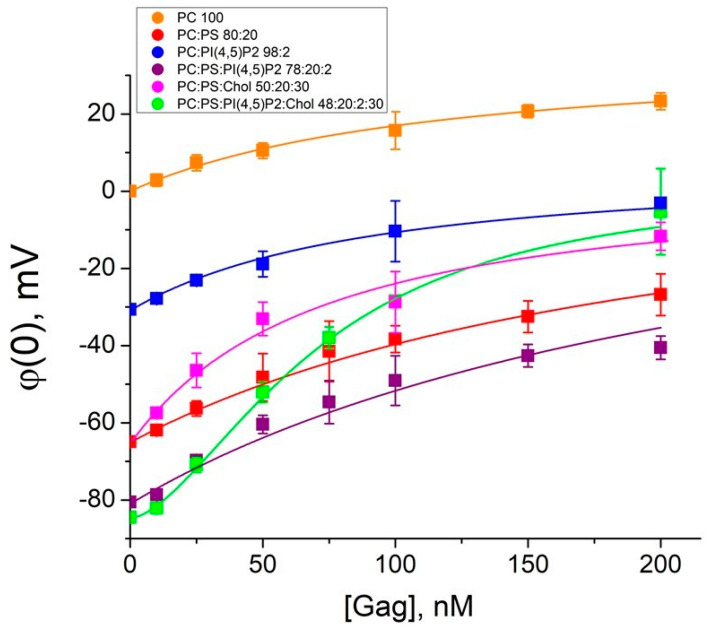
Isotherms of the Gag adsorption onto the membranes. In the legend, the ratios are given in mol% for mixtures 1–6. Fitting curves were plotted using Equation (5) for mixtures 1–5 and Equation (6) for mixture 6. Each point in the plots was obtained by averaging the results of 3–5 independent experiments. Error bars in the plots represent the standard deviation. If the error bars are smaller than the size of the circle in the plot, the error bars are not shown.

The measured values of the difference of boundary potentials on BLMs for each Gag concentration were recalculated into surface potentials taking into account the values of the ζ-potential (Figure 3). The zero corresponded to the value of the BLM surface potential before Gag adsorption.

To determine the Gag affinity to membranes, we calculated binding constants for mixtures 1–5 using Langmuir isotherm (5):

(5) $$\sigma =\frac{\sigma_{Gag}zK_{ads}C_{Gag}exp(-\frac{zF\varphi (0)}{RT})}{1+K_{ads}C_{Gag}exp(-\frac{zF\varphi (0)}{RT})}+\sigma_{0},$$

where $\sigma_{0}$ and σ are surface charge densities before and after Gag adsorption, respectively, K_ads_ is the Gag–membrane binding constant, σ_Gag_ is the surface charge density created on the membrane by the adsorbed Gag molecules (defined as 0.9 × 10^−6^ C/cm^2^ [35]), C_Gag_ is the bulk concentration of Gag, φ(0) is the surface potential, and z is the charge number.

In the case of mixture 6, the Langmuir isotherm was clearly not a good model to describe the experimental data (χ^2^ >> 1; coefficient of determination R^2^ < 0.9). Gag is able to form low-order oligomers by itself even in solutions without RNA or other cellular factors [7]. Therefore, we used Hill Equation (6), which takes into account possible cooperative protein–lipid interactions reported in the literature [16,20], to fit our measured data. The binding constants are presented in Table 2. The binding constants are of the order of 10^7^ M^−1^, which corresponds to dissociation constants (which are the inverse parameter) of the order of 100 nM (Table 2). Thus, for all lipid mixtures, we measured concentrations at least two times higher than the dissociation constant, which validates the fit by the corresponding isotherms. Unfortunately, for the Gag concentrations above 300 nM, the membranes were unstable due to the surface activity of the protein, which we have previously reported [51]. Nevertheless, the obtained values are of the same order of magnitude as the constants found for HIV-1 Gag adsorption at PI(4,5)P2-containing membranes by the technique of the second harmonic generation [57], as well as for Rous sarcoma virus (RSV) Gag adsorption at charged membranes by surface plasmon resonance [20]. Interestingly, the values of binding constants for Gag of both HIV-1 and RSV are two orders of magnitude higher than those for MA domain [16,20].

(6) $$\sigma =\frac{\sigma_{Gag}zK_{ads}^{n}({C_{Gag}exp\left(-\frac{zF\varphi \left(0\right)}{RT}\right))}^{n}}{1+K_{ads}^{n}({C_{Gag}exp\left(-\frac{zF\varphi \left(0\right)}{RT}\right))}^{n}}+\sigma_{0},$$

For membranes without cholesterol (mixtures 1–4), the binding constants were approximately the same regardless of the difference in charge density on the surface of the BLMs. This implies that the affinity of Gag to these lipid membranes is uniform, while the apparent differences in Gag adsorption were determined by the difference in Gag concentration near the surface of the BLMs. In the presence of cholesterol (mixtures 5 and 6), the binding constants were approximately twice as high, although cholesterol did not alter the total surface charge density of the membranes. A specific protein–lipid interaction was observed for the BLM of mixture 6. The type of adsorption changes and cooperative protein–protein interactions were possible (with the Hill coefficient n obtained as 1.9 ± 0.1 ≈ 2). The obtained value of the Hill coefficient suggested a low degree of protein oligomerization near the membrane surface. These data are consistent with studies demonstrating Gag monomer–dimer or monomer–trimer equilibrium in solution [4,7], and dimers/trimers on charged surfaces by atomic force microscopy [58]. The charge number *z*~1 indicated that the protein was adsorbed onto the membrane with the same density of charged amino acid residues, as lipid charges were located in the membrane.

## 4. Discussion

The interaction of the Gag polyprotein with the plasma membrane is the necessary step to initiate the viral assembly process. This involves the binding of Gag to various lipids [59]. Therefore, much attention has been paid to the study of this process. Nevertheless, quantitative data on the binding of HIV-1 Gag to lipids are scarce. Most of them are aimed at studying the specific interaction of the protein with PI(4,5)P2 [57]. PI(4,5)P2 is thought to interact specifically with the N-terminal myristoyl, leading to conformational rearrangements in the protein [15,60]. Myristoylation can enhance binding, but MA protein lacking the myristoyl moiety can still bind to membranes [16]. In addition, Gag from human sarcoma virus (another retrovirus) does not have a myristoyl moiety. Here, we investigated the affinity of Gag to lipid membranes from the point of view of electrostatic effects and specific protein–lipid interactions, excluding a myristoyl-PI(4,5)P2 interaction. Systematic variation of the membrane lipid composition allowed us to determine the intrinsic binding constants of the protein to membranes, taking into account electrostatic attraction.

Our results (Table 1) showed that the surface charge of the studied BLMs is provided predominantly by phosphatidylserine molecules taken in an amount of 20 mol%, while even 2 mol% PI(4,5)P2 is sufficient to increase the surface charge density of the membrane by ~40%. A strong effect of PI(4,5)P2 can be explained by the structure of the polar head group of this lipid, which ensures the presence of three to five anionic charges at a neutral pH of the electrolyte [48]. Cholesterol did not affect the total membrane charge density. The calculated values of membrane surface charge density were in agreement with the literature data for liposomes containing phosphatidylserine and PI(4,5)P2 [44,47,48,49].

The obtained values of the binding constants (Table 2) manifest that the preference of Gag for negatively charged membranes is due to the difference in protein concentration near the membrane surface resulting from its overall positive charge. Thus, it is a consequence of a stronger electrostatic attraction rather than a specific interaction between the protein and the lipids. In fact, prior to adsorption, protein should be present in the diffuse layer near the membrane. In this layer, the protein concentration increases according to the Boltzmann distribution, as described by the GCS theory, if the protein charge is opposite to that of the membrane. Alternatively, the protein concentration should decrease at the membrane/water interface. While the Debye length is comparable to or larger than the protein size, this distribution should not depend on the protein orientation near the membrane surface. The electrostatic attraction depends on the charge density of the membrane. The general negative charge of the membrane is provided by phosphatidylserine, which is present in the BLMs at 20 mol%. Although Gag is thought to interact specifically with PI(4,5)P2, its presence in the membrane at 2 mol% had virtually no effect on the value of the binding constant. Interestingly, Gag was able to adsorb onto the BLM of phosphocholine, demonstrating a principle possibility of interaction with uncharged membrane (in contrast, for example, to the M1 protein of influenza A virus, which was studied in the same way [39,61]). The values of the boundary potential difference are approximately the same for mixtures 1 and 3, despite the fact that the membrane surface charge increased by about 30 mV in the presence of 2 mol% PI(4,5)P2. We assumed that the protein is adsorbed on uncharged membranes not through the MA domain but through a different structural element, and the observed ~30 mV is a consequence of the charge on the MA domain facing outward from the membrane. For the charged membrane, it is oriented toward the surface of the membrane. This results in a surface potential of about 60 mV − 30 mV = 30 mV (see Table 1) for PS-containing membranes, while the presence of only 2 mol% PI(4,5)P2 did not change the protein orientation on the membranes. These estimates are additionally confirmed by the charge number values of z ≈ 1, which show that the charge density on the protein is the same as at the membrane. These findings allow us to hypothesize that the presence of RNA in solution, which is shown to be necessary for the Gag extension and correct assembly of the Gag lattice in cells, may be required for the proper orientation of the MA domain near the membrane. If negatively charged RNA is present in the cytoplasm, it should bind two positively charged lysins to the HBR of the MA domain, preventing them from interacting with the anionic lipids of the cytosolic leaflet of the PM. This may be the requirement for further extension of the Gag molecule for the assembly of the immature virion.

Recently, we have shown that Gag–membrane interactions should involve the insertion of its amphipathic helix from the CA-SP1 junction region [51]. The results of the present study support this finding. Furthermore, the specificity of Gag-PI(4,5)P2 interactions was observed only in the presence of cholesterol. We suggest that cholesterol may enhance Gag binding by promoting hydrophobic protein–lipid interactions beyond electrostatic attraction. Cholesterol did not alter the measured total surface charge density $\sigma_{0}$ but the difference in boundary potentials increased, especially in the presence of PI(4,5)P2. A more favorable interaction with cholesterol-containing membranes may result from the ability of this lipid to form clusters with acidic lipids, as reported previously [30,32,62]. Such reorganization of the membrane structure can lead to local charge changes and the formation of additional hydrogen bonds that stabilize the intramolecular attraction of lipids [30]. Therefore, this can locally alter membrane properties and, as a consequence, the nature of adsorption, demonstrating cooperative protein–protein interactions.

Our results suggest that in the presence of cholesterol, the value of the binding constant increases two-fold, demonstrating cooperative behavior in the presence of PI(4,5)P2. At the same time, cholesterol did not affect the total lipid charge density of the BLM, measured within the GCS model by the IFC method. Thus, Gag adsorption initiates the formation of PI(4,5)P2- and cholesterol-enriched domains rather than association with pre-existing microdomains [63]. This is consistent with the finding that PI(4,5)P2 is enriched in the viral envelope compared to the cell plasma membrane [64].

In summary, we can conclude that Gag–membrane binding could occur via both electrostatic and hydrophobic interactions with different protein regions involved in this process. Therefore, at least in the early stages, it does not occur solely through the MA domain and additional factors, such as the presence of RNA, may be required for the proper orientation of this domain near the membrane. This could be a reason for the reported necessity of RNA for Gag extension and formation of the immature virion. Taking into account electrochemical aspects of Gag–membrane interactions, we found that Gag–membrane binding is largely independent of lipid composition, and charged lipids only change the near-surface concentration of the protein. In contrast, cholesterol, and especially cholesterol-PI(4,5)P2 clusters, drastically alter Gag–membrane affinity and may induce Gag oligomerization. Therefore, these lipid clusters may serve as nucleation points for the formation of the Gag lattice of immature virions.

Finally, we can say that our electrochemical approach to measuring membrane boundary potentials goes far beyond the classical binding of ions and small molecules [65,66,67]. It allows the study of the binding of peripheral membrane proteins [68,69], including matrix and capsid proteins of enveloped viruses [39,49,70]. Furthermore, the IFC technique allows the separation of general electrostatic interactions of charged amino acid residues of proteins with charged lipids from specific binding. Thus, we can extract additional data on the specific reactions and important partners in protein-membrane interactions.

## Figures and Tables

**Table 1:** Electric potentials at the surface of lipid membranes.

Lipid Mixture	ζ-Potential, mV	Surface Potential, mV	Charge Density × 10^−6^ C/cm^2^
1	−3.7	−3.9	−0.09 (≈0)
2	−59.8	−64.9	−2.3 ± 0.1
3	−28.7	−30.7	−0.95 ± 0.02
4	−75.4	−82.8	−3.3 ± 0.1
5	−57.7	−62.5	−2.1 ± 0.1
6	−76.8	−84.5	−3.1 ± 0.1

**Table 2:** The binding (K_ads_) and corresponding dissociation (K_d_) constants of the Gag polyprotein to lipid membranes. χ^2^ and coefficient of determination (R^2^) represent the accuracy of the fittings.

Lipid Mixture	K_ads_, ×10^7^ M^−1^	K_d_, nM	Charge Number, *z*	Reduced χ^2^	R^2^
1	0.79 ± 0.04	127 ± 6	1.0 ± 0.1	0.07	0.998
2	0.81 ± 0.04	123 ± 6	1.11 ± 0.03	0.16	0.994
3	1.3 ± 0.2	77 ± 12	1.21 ± 0.02	0.29	0.994
4	0.9 ± 0.1	111 ± 12	1.12 ± 0.03	0.79	0.987
5	2.1 ± 0.2	48 ± 5	1.0 ± 0.1	0.74	0.989
6	2.5 ± 0.1	40 ± 2	1.02 ± 0.01	0.37	0.998

## Data Availability

Data will be available upon reasonable request.
